# Development of Nanosuspension of *Artemisia absinthium* Extract as Novel Drug Delivery System to Enhance Its Bioavailability and Hepatoprotective Potential

**DOI:** 10.3390/jfb14080433

**Published:** 2023-08-18

**Authors:** Nazish Jahan, Fareeha Kousar, Khalil Ur Rahman, Syeeda Iram Touqeer, Naseem Abbas

**Affiliations:** 1 Natural Products Lab, Department of Chemistry, University of Agriculture, Faisalabad 38000, Pakistan; nazishjahan@uaf.edu.pk (N.J.); fareehakousar87@gmail.com (F.K.); iram_touqeer@yahoo.com (S.I.T.); 2 Department of Biochemistry, Riphah International University, Faisalabad 38000, Pakistan; khaliluaf@yahoo.com; 3 Department of Mechanical Engineering, Sejong University, Seoul 05006, Republic of Korea

**Keywords:** *Artemisia absinthium*, nanosuspension, response surface methodology, bioavailability

## Abstract

A nanosuspension of *Artemisia absinthium* extract was formulated and characterized for the enhancement of bioavailability and better hepatoprotective efficacy. The nanosuspension of *A. absinthium* extract was formulated using an antisolvent precipitation technique, and various formulation parameters were optimized using response surface methodology (RSM). The optimized nanosuspension was characterized using AFM and FT–IR spectroscopy. The drug-release profile and oral bioavailability of the optimized nanosuspension were assessed with reference to coarse suspension. The DPPH radical scavenging method was used to measure the nanosuspension’s antioxidant activity, and its in vivo hepatoprotective potential was assessed against CCl4-induced hepatic injury in rats. The developed optimized nanosuspension had suitable zeta potential of −11.9 mV, PDI of 0.285, and mean particle size of 253.8 nm. AFM study demonstrated a homogeneous population of nanoparticles with average size of 25 nm. The formulated nanosuspension of *A. absinthium* showed faster dissolution rate and 1.13-fold enhanced bioavailability as compared to the coarse suspension (plant extract). Furthermore, the nanoformulation had stronger antioxidant and hepatoprotective potential as compared to the unprocessed coarse extract. These results demonstrated that nanosuspension is a promising strategy for improving the oral bioavailability and bioactivities of *A. absinthium* extract.

## 1. Introduction

Recently, the use of medicinal plants has increased all over the world due to their promising health benefits and safety [1,2]. The World Health Organization (WHO) is giving prime importance to the evaluation of secondary metabolites derived from natural products for the treatment of diseases due to broad spectrum management [3]. About 80% of the population in developing countries uses natural products for their primary health needs [4]. Wormwood, also known as *Artemisia absinthium* L., is a significant perennial shrubby medicinal plant that is indigenous to Asia, the Middle East, Europe, and North Africa [5]. *A. absinthium* exhibits several pharmacological activities including antimalarial [6], antimicrobial, antioxidant, anti-inflammatory [7], and nephroprotective activities, as well as an excellent hepatoprotective potential [8]. Aerial parts are used in gastric herbal preparations, in dietary supplements, and in alcoholic beverages [9]. Various phytochemicals such as essential oil, absinthin, anabsinthin, anabsin, lactones, and organic acids have been reported in *A. absinthium* extract. It also possesses flavonoids such as quercetin, myrecetin, hesperidin, rutin [10], and artemisnin and phenolic acids such as salicylic, coumaric, syringic, vanillic, and chlorogenic acids, which are probably involved in the mechanism of free-radical scavenging activity [11]. Many medicinal activities of *Artemisia* are reported, but the formulation of nanosuspension and pharmacokinetic studies of *A. absinthium* extract are reported for the first time in this study.

It is evident that most of the bioactive constituents of herbal extracts are unable to cross the lipid membrane due to their large molecular diameter and low water solubility that further results in poor systemic absorption and low bioavailability [12,13]. Nanotechnology has recently emerged as a novel technique to address the difficulties of solubility and bioavailability of poorly water-soluble pharmaceuticals. The herbal extracts processed through nanotechnology may enhance the efficacy of bioactive phytopharmaceuticals by increasing their water solubility and bioavailability as well as by reducing the treatment dose and side effects [14]. Various novel drug delivery systems like polymeric nanoparticles, liposomes, nano emulsions, and phytosomes for plant extracts and their bioactive compounds have been reported [15]. Formulation of nanosuspension is an engrossing strategy for increasing the dissolution velocity and bioavailability of synthetic drugs and phytochemicals due to the increased surface area of nanoparticles. [16]. The medication delivery method known as nanosuspension uses nanosized drug particles that are stabilized by polymers or surfactants [17,18]. Pharmaceutical nanosuspension of herbal medicines is a bewildering and fascinating strategy because it increases saturation solubility, and it poses fewer side effects with high drug loading. Moreover, various administration routes of oral, parenteral, intravenous, and pulmonary delivery systems can be applied to nanosuspensions. A variety of formulation techniques, such as micronization, solid dispersion, inclusion complexes, lipid carriers and liposomes, and emulsions and micro-emulsions are extensively used to overcome the solubility problem of poorly soluble drugs. However, the main difficulty associated with these approaches is their lack of universal applicability to all kinds of drugs. Nanosuspension technology has revealed greater potential to resolve the problem because of simplicity and the advantages it bestows over other techniques. Pharmaceutical nanosuspensions are extremely fine, dispersed solid drug particles in an aqueous phase stabilized by surfactants, polymers, or a mixture of both. The size of nanoformulated drug particles is usually smaller than 1 micrometer with an average size ranging from 200 nm to 600 nm. This formulation has low processing cost, high dug loading, and diminutive side effects for excipients. Owing to the improved surface-to-volume ratio of nanosuspensions, drug particles exhibit greater saturation solubility and faster dissolution rates, which ultimately improve bioavailability [18]. Therefore, the goal of the current research was to formulate an *A. absinthium* extract nanosuspension with increased oral bioavailability and dissolution.

## 2. Materials and Methods

### 2.1. Plant Extract Preparation

The aerial portions of *Artemisia absinthium* were purchased from a local market in Faisalabad and identified from a plant taxonomist from the Department of Botany, University of Agriculture, Faisalabad. N-hexane was used to remove the fat of the plant material. Plant ethanol extract was prepared using the Soxhlet device. Briefly, 30 g of defatted plant material was introduced to the Soxhlet extractor’s thimble, and 300mL of ethanol (99.9%, MERCK Germany, Darmstadt, Germany) was taken in flask and heated for 8 h. The resulting ethanolic extract was filtered and concentrated on Rotary evaporators (Buchi, CH-9230 Flawil 1, Flawil, Switzerland) at low temperature (40 °C) and pressure (50 torr). The concentrated ethanolic extract was kept in the refrigerator (2–8 °C) for additional examination. 

### 2.2. Formulation and Optimization of Nanosuspension

*A. absinthium* extract was made into a nanosuspension utilizing the antisolvent precipitation method reported by Thadkala et al., 2014, with some modification. Plant extract (0.25 g) was thoroughly diluted in 10mL ethanol (*w*/*v*) (99.9%, MERCK, Germany); then this organic solution was slowly injected (1 mL/min) with the help of a syringe connected to a thin Teflon tube into aqueous phase containing hydroxypropyl methylcellulose (HPMC) (K4M, Sigma-Aldrich Germany, Taufkirchen, Germany) with continuous mechanical stirring at 6000 rpm for 6 h at room temperature [19]. RSM was used to optimize various nanosuspension parameters. Average particle size, poly dispersity index (PDI), and zeta potential were the study’s response variables, whereas stabilizer-to-plant extract ratio, antisolvent/solvent ratio, and stirring time were its input components. Stabilizer-to-plant extract ratio (*w*/*w*) varied from 0.20:1 to 1:1, antisolvent/solvent ratio (*v*/*v*) from 5:1 to 15:1, and stirring time was varied from and 2 to 6 h. The design of the experiment was used to optimize the selected parameters. Based on the number of factors and their levels, central composite design (CCD) was used to investigate the effects of various parameters on physical properties of prepared nanosuspensions. The design consists of a total of 20 experiments and data obtained via these experiments were analyzed using Design Expert software (Minneapolis, MN, USA). Optimized nanosuspensions were lyophilized at −60 °C for 72 h. Freeze-dried samples were ground to fine powder and used for solid-state characterization.

### 2.3. Stability of Nanosuspension

At two different temperatures, the physical stability of the optimized nanosuspension was assessed. For this use, the prepared nanosuspension was kept at 4 °C in the refrigerator for three months and at room temperature between 25 and 30 °C. Aliquots of samples were taken after three months, and their particle size, PDI, and zeta potential were all examined.

### 2.4. Characterization of Nanosuspension

Freshly prepared nanosuspension particle size, PDI, and zeta potential were measured using the DLS (Dynamic Light Scattering) technique and the Zetasizer (Nano ZS, Malvern Instruments, Malvern, UK). The optimized nanosuspension surface morphology and topography were assessed using a scanning electron microscope (JEOL, JSM-6400, Tokyo, Japan) at various magnifications. An atomic force microscope (AFM Shimadzu WET-SPM 9600, Tokyo, Japan) was used to obtain three-dimensional (3D) pictures of the optimized nanosuspension. On an FT–IR spectrometer (Perkin Elmer Spectrum, Version 10.4.3, USA), the molecular interactions of stabilizer-optimized nanosuspensions were captured. For this, FT–IR analysis was performed on HPMC (stabilizer), designed nanosuspension, and coarse plant extract. The spectra were collected from the KBr disc. A small amount of material was applied to the lens, and scans were made with a 4000–450 cm^−1^ scanning range.

### 2.5. Dissolution Investigation

The optimized nanosuspension dissolution profile with reference to coarse extract were determined in phosphate buffer using USP dissolution Type-II (pharma test de, ISO 9001, Germany). Equivalent amounts of lyophilized powder of optimized nanosuspension and coarse plant extract were poured into empty gelatin capsules. The dissolution medium was phosphate buffer (pH 7.4, 900 mL) at 37 °C, and the rotation speed was 100 rpm. An amount of 10 mL of the dissolution liquid were removed at predetermined intervals (10, 20, 30, 50, 60, 90, and 120 min), and the amount of medication dissolved was determined spectrophotometrically at 237 nm using quercetin (≥95%, HPLC grade, Merck Germany) as the standard compound. The percentage drug release of nanosuspension was compared with coarse suspension. Results of the dissolution test, which was carried out in triplicate, are expressed as Mean ± SD (n = 3) [20]. 

### 2.6. Pharmacokinetic Study

Healthy Sprague Dawley rats weighing 250–300 g were used as experimental rats to perform pharmacokinetic studies of nanosuspensions. Before doing the experiments, the animals were fed a regular diet for one week. The rats fasted for twelve hours before the experiment but with free access to water. The coarse extract and nanosuspension at doses of 50 mg kg^−1^ were orally administered to rats. Blood samples of about 0.5 mL were collected using cardiac puncture under mild anesthesia into heparinized tubes at 0.5 h, 1 h, 2 h, 4 h, 6 h, and 24 h time intervals. For separation of plasma, the collected blood samples were centrifuged at 4000 rpm for ten minutes. All plasma samples were stored at −20 °C for further analysis [21].

#### HPLC Analysis

For extraction of quercetin, plasma (200 µL) was mixed with 400 µL methanol (99.9%, HPLC grade, Merck, Germany) and HCl (200 µL, 25%, analytical grade, Merck, Germany). The resulting mixture was vortexed in a vortex shaker for 90 s and incubated at 50 °C for fifteen minutes [22]. Then, this mixture was centrifuged at 10,000 rpm for ten minutes. The supernatant (20 µL) was injected into the column for determination of quercetin in plasma. The mixture of Na_2_HPO_4_ (≥99%, HPLC grade, Merck, Germany) (30 mM), acetonitrile (99.9%, HPLC grade, Merck, Germany), and methanol (65:29:6, *v*/*v*/*v*) was used as a mobile phase at the flow rate of 1 mL min^−1^. The column effluent was analyzed at 370 nm using a UV–Visible detector. The standard plots of quercetin were constructed, and pharmacokinetic parameters were determined using MS Excel, 2010. Maximum peak concentration in plasma (C_max_), the time required for maximum concentration (T_max_), was determined from concentration time curves. Area under curve (AUC) was determined using the trapezoidal method. Results were expressed as Mean ± SD (n = 3).

### 2.7. In Vitro Antioxidant Activity

In vitro antioxidant activity of the coarse extract (ethanolic extract suspended in water) and nanosuspension were evaluated via 2, 2-Diphenyl-1-picrylhydazyl (DPPH) radical scavenging method [23,24]. Five concentrations of nanosuspension and coarse extract ranging from 0.02 to 0.1 mg/mL were made. Then, 1 mL of freshly prepared DPPH (99% Merck, Germany) solution (0.1 mM in methanol) was added to 3 mL of each sample solution, and the reaction mixture was incubated in the dark for half an hour. The absorbance of the reaction mixture was measured at 517 nm after incubation. Ascorbic acid was utilized as the standard, and DPPH solution as the blank. The decrease in absorbance indicated that plant extracts inhibited the DPPH radical to a larger extent. All experiments were carried out in three replicates, and the average results were provided. Percent inhibition of DPPH free radical was calculated using formula given below:

Inhibition of DPPH free radical (%) = A_c_ − A_s_/A_c_ × 100

where A_c_ represents absorbance of control, and A_s_ represents absorbance of samples. 

### 2.8. In Vivo Hepatoprotective Potential 

The in vivo hepatoprotective potential study was carried out in accordance with international ethical guidelines and was overseen by a veterinarian at the Clinical Medicine and Surgery Department, UAF. The proposal was approved by the synopsis scrutiny committee of the Department of Chemistry, University of Agriculture, Faisalabad and endorsed by the graduate research board through letter No. DGS-20525-28. In this investigation, healthy Sprague Dawley rats of either sex weighing 200–250 g were employed. Animals were housed in the Clinical Medicine and Surgery department of the University of Agriculture in Faisalabad. Rats were acclimatized for seven days before starting the experiment. 

For evaluation of hepatoprotective potential of nanosuspensions and plant extract, hepatotoxicity in rats was induced via oral administration of CCl_4_ (1 mL/kg body weight) for two consecutive days. After inducing hepatotoxicity, various curative treatments were given to these CCl_4_-intoxicated rats. The curative hepatoprotective potential of plant extracts were compared with nanosuspension.

#### Experimental Design 

Rats were randomly divided into different treatment groups with four rats in each group. The detailed treatment protocol was as follows:

Group I: (normal control) received a normal diet during all the experiment.
Group II: (positive control) rats were orally administrated with CCl_4_ at the dose 1 mL/kg body weight mixed with olive oil (1:1).
Group III (CCl4 + AA NS): Rats were orally administered CCl_4_ to induce liver toxicity. Then these rats received nanosuspension at dose of 100 mg/kg for four consecutive days. Blood samples were collected daily after twenty-four hours of each treatment.
Group IV (CCl4 + AA CE): These rats were orally administered carbon tetrachloride (1 mL/kg body weight) for two consecutive days to induce liver toxicity. After that, plant extract (150 mg/kg body weight) was given to these CCl_4_-intoxicated rats for four days. Blood samples were collected after twenty-four hours of each curative treatment.

After collection, blood samples were centrifuged at 3000 rpm, and serum was analyzed for different biochemical parameters such as aspartate aminotransferase (AST), alanine aminotransferase (ALT), alkaline phosphatase (ALP), albumin (Alb), total protein (TP), and total bilirubin (TB) using a chemistry analyzer (Semar-S1000 elite, Prato, Italy). On the last day of experiment, all the animals were slaughtered, and liver tissues were removed and washed with buffered saline. Liver homogenate was prepared by homogenizing the liver tissues in ice-cold phosphate buffer (pH 7.4). Superoxide dismutase (SOD), catalase (CAT), and glutathione peroxidase (GPX) were among the antioxidant enzymes measured in the homogenate mixture after centrifugation at 10,000 rpm 

### 2.9. Statistical Analysis

CCD was used to improve the parameters of the nanosuspensions, and Design Ex-pert (version 7.1, Stat-Ease, Inc., Minneapolis, MN, USA) was utilized to examine the data. The statistical difference in means was analyzed using ANOVA, followed by Tukey’s test, and *p* < 0.05 was considered statistically significant. All data were reported as Mean ± SD.

## 3. Results and Discussion

### 3.1. Optimization of Nanosuspension

In the present study, CCD (central composite design) was used to study the effect of various formulation variables on particle size, PDI (polydispersity index), and zeta potential. The experimental data of all experimental runs with response variables are given in Table 1. The ranges of three responses Y_1_, Y_2_, and Y_3_ were found to be 224.3 nm to 605 nm, 0.295 to 0.590, and −2.00 mV to −24.50 mV, respectively. The responses obtained via experiments were treated with linear, cubic, and quadratic model functions to select the best fitting models for optimization study. The quadratic model was chosen in the end to establish the mathematical connection between the input variables and the results. Analysis of variance (ANOVA) was used to determine the importance of the selected model. The coefficient of determination (R^2^) is an important statistical parameter used to check the fitness of a model. A high value of R^2^ indicates a significant correlation between observed and predicted values of response [25]. The values of R^2^ for particle size, PDI, and zeta potential are all 0.9963, 0.9416, and 0.8896, respectively, demonstrating strong correlations between experimental and predicted values. The polynomial regression equations in terms of coded factors describing the relationship between the input variables and response variables were also generated. The positive sign with a factor indicates increase in the response variable with the factor and vice versa.

Particle size (nm) = + 349.09 − 86.71A − 48.21B − 69.16C + 51.09AB + 0.16AC − 12.69BC + 26.18A^2^ + 4.37B^2^ + 37.39C^2^ (1)

PDI = + 0.47 − 0.081A − 0.053B − 0.064C + 0.055AB + 0.064AC − 0.022BC + 3.574E − 003A^2^ + 0.023B^2^ + 0.040C^2^ (2)

Zeta potential (mV) = + 10.73 − 1.42A + 0.48B − 0.013C − 0.013AB + 0.71AC + 0.74BC + 1.30A^2^ + 0.16B^2^ − 0.19C^2^ (3)

#### Effect of Formulation Variables

**Figure 1:**
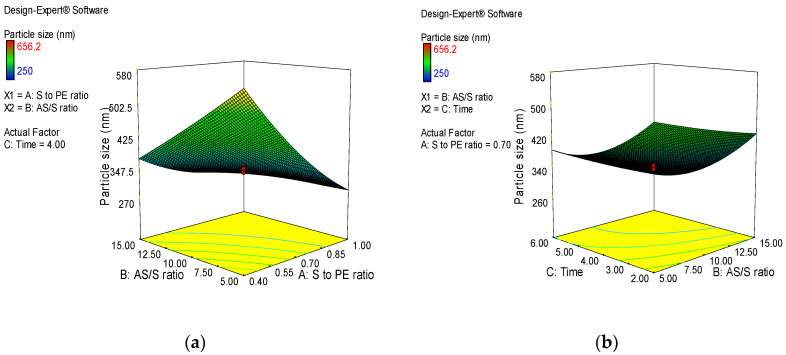
3D plots showing the combined effect of formulation variables on particle sizes of *A. absinthium* nanosuspensions. (**a**) the interaction effect of stabilizer-to-extract ratio and AS/S ratio (**b**) the interaction effect stirring time and stabilizer-to-extract ratio.

Three-dimensional (3D) response surface plots were generated to study the main and interaction effects of formulation variables on response variables. Figure 1a illustrates the interaction effect of stabilizer-to-extract ratio and AS/S ratio at a fixed level of factor C. The graph shows that the particle size was significantly decreased by increasing the stabilizer-to-extract ratio and AS/S ratio. During nanonization, the reduction in particle size resulted in tremendous increase in the surface area. The process of coverage of new surfaces with stabilizer competes with agglomeration of nanoparticles. At a higher stabilizer-to-drug ratio, more molecules of stabilizer would be able to shield the newly forming nanoparticles, which prevents agglomeration [26]. In this study, nanosuspension prepared with HPMC showed the best stability and suitable particle size. HPMC is a nonionic polymer provided with a hydrodynamic boundary layer around the nanoparticles, which prevents the nanoparticles from agglomeration [27]. The hydroxypropyl and methoxy groups of HPMC can form hydrogen bonds with polyphenols and flavonoids molecules present in *A. absinthium* extract, and the strong adsorption of HPMC on the surface of the nanoparticles inhibits the particle crystal growth and aggregation [23]. At a constant AS/S ratio, increasing the stirring time and stabilizer/extract ratio considerably reduced particle size (Figure 1b). The reduction in particle size of nanosuspensions caused by increasing stirring time revealed that longer stirring times were superior in preventing crystal formation and aggregation.

**Figure 2:**
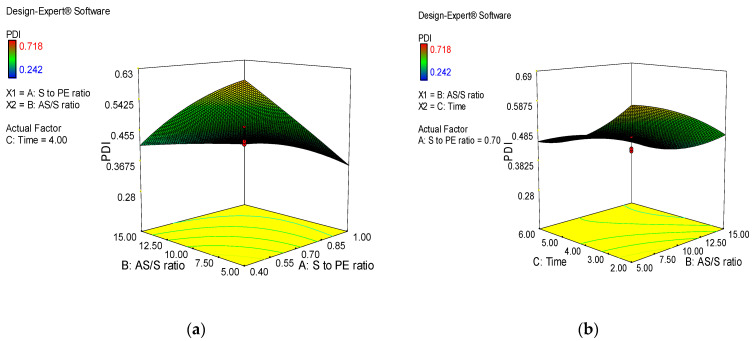
3D plots showing the combined effect of formulation variables on PDI of *A. absinthium* nanosuspensions. (**a**) the interaction effect of stabilizer-to-extract ratio and AS/S ratio (**b**) the interaction effect stirring time and stabilizer-to-extract ratio.

**Figure 3:**
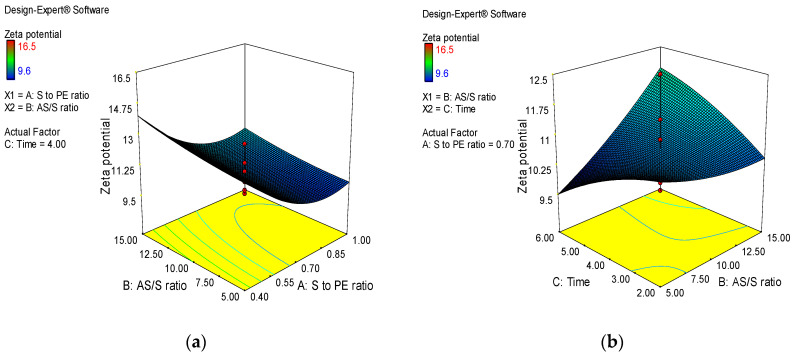
3D plots showing the combined effect of formulation variables on zeta potential of *A. absinthium* nanosuspensions. (**a**)the interaction effect of stabilizer-to-extract ratio and AS/S ratio (**b**) the interaction effect stirring time and stabilizer-to-extract ratio.

The influence of selected parameters on PDI of *A. absinthium* nanosuspensions are displayed in Figure 2. At fixed level of factor C, the interaction effect of stabilizer-to-extract ratio and AS/S ratio was found to be significant in the formulation of homogeneous particle size distribution as a sharp decreasing trend in PDI was observed by increasing the stabilizer-to-extract ratio and AS/S ratio (Figure 2a). The decreasing trend in PDI was also observed by increasing stirring time and stabilizer-to-extract ratio at a fixed AS/S ratio (Figure 2b). The observed reduction in PDI indicates that a greater amount of stabilizer with longer stirring favored the production of more uniform particle size distribution. Zeta potential is the magnitude of surface charge. It is an index of stability of a nanosuspension system [28]. High values of zeta potential are required for providing repulsion between nanoparticles with a similar charge to prevent them from aggregation [29]. The zeta potential values were significantly decreased with increasing factor A, which indicated that the adsorption layer of steric stabilizer on the surface of nanosized particles increases the distance to the plane of shear at which the zeta potential was measured (Figure 3a). 

**Figure 4:**
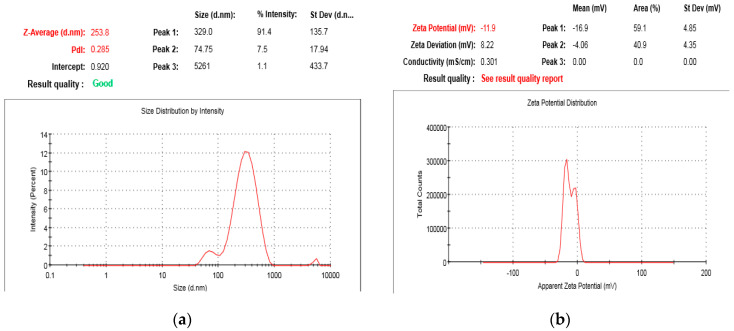
Particle size, PDI (**a**), and zeta potential (**b**) of *A. absinthium* optimized nanosuspensions.

For optimization of *A. absinthium* nanosuspensions, constraints were applied on independent response variables. In the current study, optimization was based upon minimum particle size, lower PDI, and maximization of zeta potential. The optimum values of input variables obtained via software were 1:1 of A (stabilizer-to-extract ratio), 15:1 of B (AS/S ratio), and 6 of C (stirring time) to attain the required values of response Y_1_ (251.18 nm), Y_2_ (0.294), and Y_3_ (−12.5) in the optimized formulation of *A. absinthium* extract. The fresh formulation was prepared and evaluated. The optimized formulation has a PDI of 0.258, a zeta potential of −11.9, and an average particle size of 253.8 nm (Figure 4). The observed values of the responses (dependent variables) were discovered to be in strong agreement with the values projected by the model. These findings supported the chosen model’s accuracy in predicting how input variables would affect response variables.

### 3.2. Stability Studies

After storage at 25 °C and 4 °C (for 3 months), the stability of the A. absinthium extract formulation was assessed. Over the course of three months at two temperatures, no visible deposition of particles was seen. A slight increase in average particle size and PDI values were observed at both temperatures after the storage period (Table 2). The results showed that the optimized nanosuspension of *A. absinthium* extract was physically stable due to presence of HPMC, which acts as particle growth inhibitor by providing steric repulsion. 

### 3.3. Characterization of Nanosuspension

#### 3.3.1. Atomic Force Microscopy 

**Figure 5:**
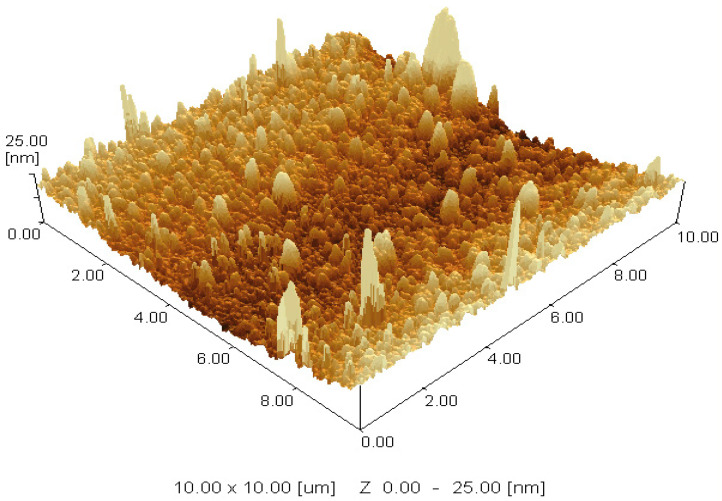
AFM image of *A. absinthium* nanosuspension.

An AFM analysis of *A. absinthium* nanosuspension revealed that particles were uniform with an approximate size of 25 nm. The AFM 3D topography image clearly indicated a well-defined homogeneous population of nanoparticles with good surface characteristics (Figure 5). The particle size measured using AFM is smaller than that with Zeta sizer (DLS). Particle size variations may be observed when using different methodologies to measure the size of nanoparticles. The difference between the diameters evaluated using the two approaches is clear since AFM examines the physical diameter of the nanoparticles, and DLS evaluates hydrodynamic diameter. Before the measurement of particle size using AFM and DSL, the lyophilized nanosuspension was suspended in distilled water and sonicated to obtain a homogenous suspension. During this process, nanoparticles may dissolve. The size measurement using DLS involved a solvation shell, so the size measurement using DLS should be a little bit greater than AFM. Solvation of nanoparticles is the reason for the greater particle size measured using DLS [30]. When lyophilized nanosuspension is suspended in distilled water and sonicated to create a homogeneous suspension prior to the determination of particle size, nanoparticles may aggregate. The inclusion of water during the lyophilization process may have caused aggregate flaws by promoting Ostwald ripening. Atomic force microscopy (AFM) demonstrated the successful preparation of plant nanosuspensions with nanoscale particle size and acceptable shape. To characterize the formulated nanosuspensions and identify their precise particle size and morphology, atomic force microscopy (AFM) is preferred [31]. 

#### 3.3.2. FT–IR Spectra

**Figure 6:**
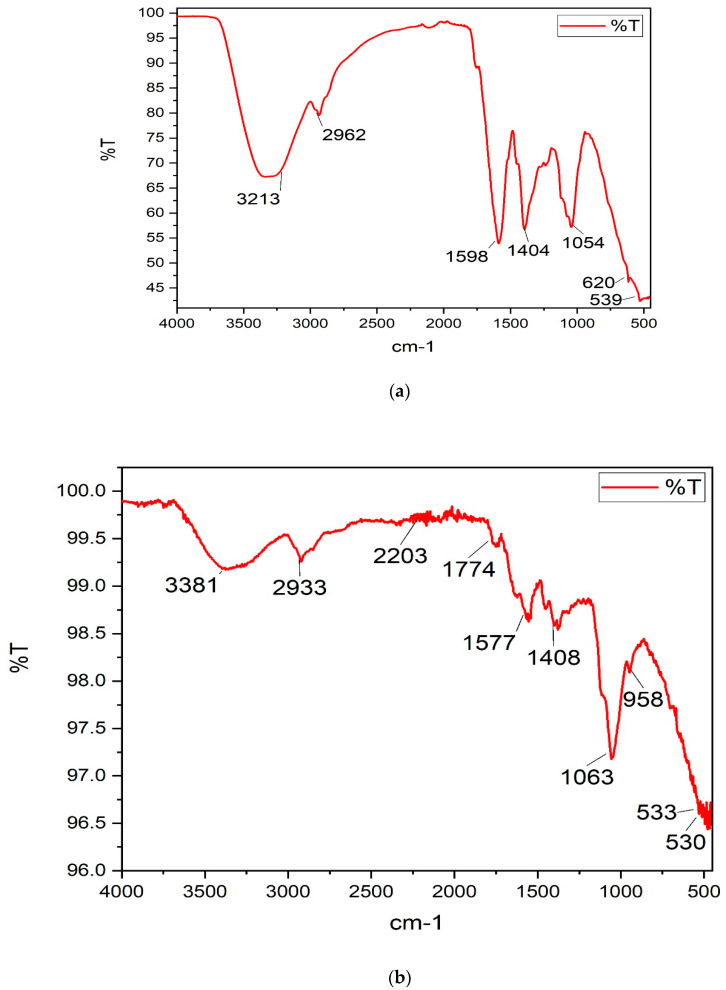

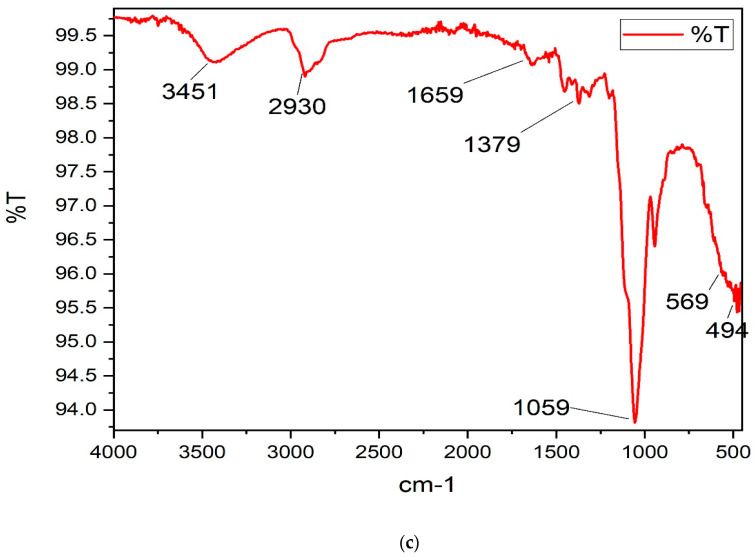
(**a**) FT–IR spectrum of *A. absinthium* extract. (**b**) FT–IR spectrum of *A. absinthium* nanosuspension. (**c**) FT–IR spectrum of HPMC.

The FT–IR spectra of *A. absinthium* extract, *A. absinthium* nanosuspension, and HPMC (stabilizer) are compared in Figure 6a–c. The spectrum of *A. absinthium* coarse extract presenting a strong and broad band at 3213 cm^−1^ was assigned to the stretching vibrations of the –OH group of flavonoids. This peak shifted to 3381 cm^−1^ and became less broad in the nanosuspension, which revealed inter-molecular hydrogen bonding between the phenolic -OH group and HPMC. The peaks observed at 1598 cm^−1^ (C=C aromatic stretching) and 1404 cm^−1^ (–C-H bending of aromatic hydrocarbon) in *A. absinthium* extract shifted to 1577 cm^−1^ and 1408 cm^−1^, respectively, in the nanosuspension spectrum. Other peaks in the *A. absinthium* extract were observed at 2962 cm^−1^, 1054 cm^−1^, and 620 cm^−1^. However, these peaks were not found in the spectrum of *A. absinthium* nanosuspension. These results indicated that during formulation of nanosuspension some sort of interaction developed between plant phytoconstituents and the stabilizer. 

### 3.4. Dissolution Study 

**Figure 7:**
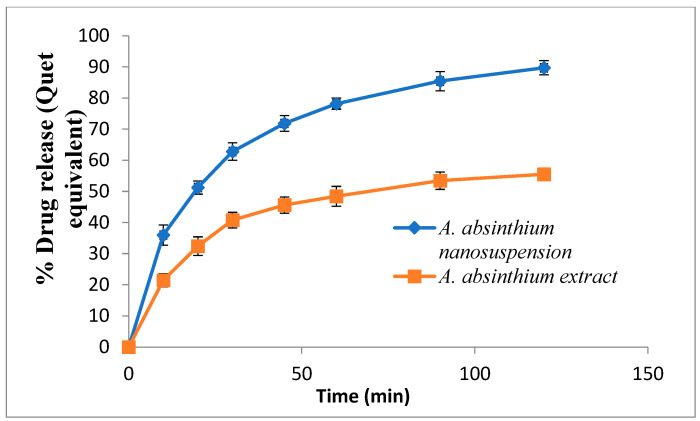
*A. absinthium* nanosuspension and coarse extract dissolution profiles. The results are presented as Mean ± SD (n = 3).

The dissolution rate of the nanosuspension of *A. absinthium* was compared with the coarse extract by measuring the concentration of quercetin, and results are depicted in Figure 7. The dissolution medium was phosphate buffer (pH 7.4, 900 mL) at 37 °C. The release profile of the *A. absinthium* extract showed slow dissolution with 21.45% release within the first ten minutes. On the other hand, the *A. absinthium* nanosuspension indicated faster dissolution as 35.96% quercetin was released within the first ten minutes. After complete dissolution (within 120 min), the amount of quercetin released from the *A. absinthium* nanosuspension (89.71%) was still greater than the *A. absinthium* coarse extract (55.50%). The dissolving velocity of the nanosuspension was dramatically accelerated by the smaller particle size and increased surface area of nanoparticles [32,33]. Due to the presence of surface stabilizers, poorly soluble flavonoids in plant extract have higher solubility and wettability in the dissolving medium, which could also account for the increased dissolution rate of nanosuspensions [34]. 

### 3.5. Pharmacokinetic Study 

**Figure 8:**
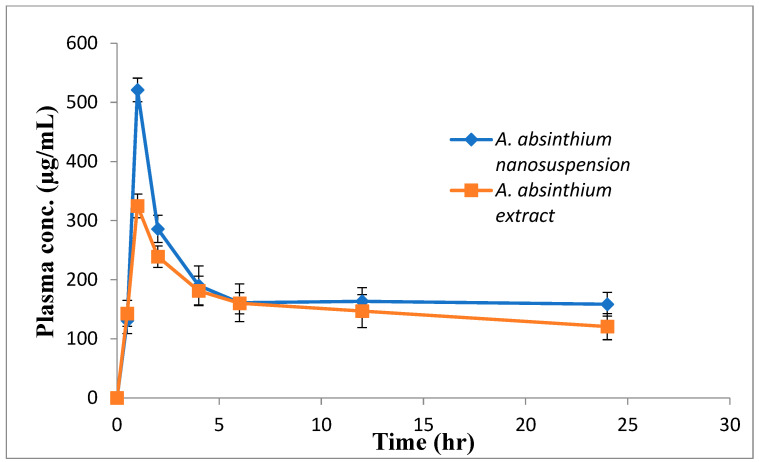
Plasma concentration–time curves after oral administration of *A. Absinthium* nanosuspension and coarse extract (quercetin equivalent) to rats. Results are expressed as Mean ± SD (n = 3).

Figure 8 displays the mean plasma concentration versus time curves following oral administration of *A. absinthium* nanosuspension and coarse extract at doses of 50 mg/kg (with reference to quercetin). Plants contain many bioactive phytoconstituents, and it is immensely difficult to evaluate the concentration of all these constituents, so in the ongoing research only one key bioactive component was used as the standard compound to compare results. For this purpose, quercetin, which is the major constituent of Artemisia extract, was used as the standard compound to evaluate the dissolution profile as well as for pharmacokinetic studies of nanosuspensions. The dissolution profile of the *Artemisia* extract and nanosuspension was expressed as “quercetin equivalent”. After 1 h of oral administration, the value of C_max_ of *A. absinthium* nanosuspension (521.2 µg/mL) was significantly (*p* < 0.05) higher than coarse extract (324.93 µg/mL). The value of AUC_0–24h_ of nanoformulation was 1.13-fold greater than coarse extract. In the present investigation, the larger C_max_ and AUC values indicated improved absorption of the nanosuspension. Nanosuspension technology significantly increased the saturation solubility and dissolution rate due to smaller particle size and greater surface area results in enhanced bioavailability [35,36]. In addition, nanoparticles exhibited increased bio adhesion to intestinal villi that might prolong the retention time of nanosuspensions in the gut and enhance the passive absorption [37]. 

### 3.6. In Vitro Antioxidant Activity

Antioxidant potentials of nanosuspensions and coarse extract were determined using the DPPH radical scavenging method. The DPPH test based on the ability of DPPH to decolorize via antioxidants is a reliable and easy method for determining radical scavenging potential of pure compounds and plant extracts [32]. The results of antioxidant potential were represented in terms of IC50 value (Table 3). Ascorbic acid, a natural antioxidant, was utilized as a control molecule and demonstrated the highest antioxidant activity with an IC50 value of 125.94 g/mL. The nanosuspension of *A. absinthium* revealed a stronger DPPH radical scavenging potential as indicated by its lower IC50 value (196.93 µg/mL) compared to the coarse extract (248.32 µg/mL). 

### 3.7. In Vivo Hepatoprotective Activity

Rats given CCl4 revealed a large (*p* < 0.05) increase in AST, alanine transaminase (ALT), ALP, and total bilirubin levels, as well as a significant (*p* < 0.05) decrease in albumin and total protein levels, confirming the presence of hepatic injury. Significant liver damage was induced by CCl4 administration, as shown by rising levels of marker enzymes in the serum. After cellular I jury, these enzymes are typically present in the cytoplasm and released into the blood. The raised levels of serums AST and ALT are highly sensitive and specific clinical biomarkers of hepatotoxicity and represent damage to the hepatocytes. Another crucial indicator of liver health is ALP, which is also widely used to assess the integrity of plasma membranes. Pathological alterations in biliary flow were detected via an elevated level of ALP in the serum. Additionally, mice treated with CCl4 had considerably lower levels of total protein and albumin. Low levels of total proteins suggested hepatic disease, as protein synthesis is a function of a healthy liver. Low albumin levels are a sign of hepatotoxicity. 

Treatment with *A. absinthium* nanosuspension at 100 mg/kg significantly reduced the activities of AST (56.24 ± 0.46 IU/L), ALT (29.27 ± 0.21 IU/L), and ALP (242.39 ± 8.25 IU/L) as well as the level of total bilirubin (0.52 ± 0.25 mg/dL) as compared to the positive control group after four days of treatment. The decreased levels of AST, ALT, ALP, and total bilirubin suggest a hepatoprotective potential of *A. absinthium* nanosuspension. Moreover, average levels of albumin (3.85 ± 0.26 g/dL) and total protein (8.78 ± 0.38 g/dL) were significantly improved in rats treated with 100mg/kg of *A. absinthium* nanosuspension. It was observed that the ameliorative effect of *A. absinthium* nanosuspension at 100 mg/kg was comparable to 150 mg/kg of *A. absinthium* coarse extract (Table 4).

The hepatoprotective activity of *A. absinthium* extract and nanosuspension could be explained due to presence of polyphenols and flavonoids, which exert strong antioxidant activity and are involved in hampering the formation of toxic metabolites of CCl_4_. Quercetin has been reported as a promising hepatoprotective compound due to its antioxidative, anti-inflammatory, and anti-apoptotic activities against various liver disorders [33]. The findings are consistent with earlier research that found quercetin, the primary bioactive component of hydroalcoholic extract flavonoid C. spinosa, to be hepatoprotective against tertiary butyl hydroperoxide (t-BHP)-induced liver damage [34]. These results indicated that *A. absinthium* nanosuspension at 100 mg/kg showed equivalent hepatoprotective activity as 150 mg/kg of *A. absinthium* coarse extract. The enhanced hepatoprotective activity of *A. absinthium* nanosuspension was due to improved bioavailability, which is in line with the results of dissolution and pharmacokinetic studies.

#### Antioxidant Enzymes

A remarkable (*p* < 0.05) reduction in activities of GPx, CAT, and SOD were observed in CCl_4_-treated rats as compared to normal healthy rats. Treatment with *A. absinthium* nanosuspension at 100 mg/kg restored the levels of GPx, CAT, and SOD, which was comparable to 150 mg/kg of *A. absinthium* coarse extract (Table 5). The in vivo antioxidant potential of *A. absinthium* nanosuspension directly linked to the flavonoids like quercetin, rutin, apigenin, and myricetin. These flavonoids have potent antioxidant activity that prevents the harmful effects caused by reactive oxygen species by neutralizing the free radicals and elevating the activities of antioxidant enzymes [38].

*A. absinthium* extract has been reported to possess antioxidant and antiparasitic activities, and its aqueous methanolic extract provides hepatoprotection against CCl_4_ and acetaminophen-induced liver injury [39]. *A. absinthium* was used to treat epilepsy, urinary disorders, and gastric problems and for wound healing. However, most of the phytochemicals such as quercetin and myrecetin suffered poor water solubility and low bioavailability [40,41,42]. In the present work, a nanosuspension of *A. absinthium* extract was developed to enhance bioavailability and bioactivity. No other research is available on the formulation of nanosuspensions of *A. absinthium* extract. In recent years, nanosuspensions have proven a promising strategy for the delivery of poorly water-soluble drugs and phytoconstituents as it increases solubility and dissolution of drug molecules. The reduced particle size offers high surface area, which dramatically increases the saturation solubility and dissolution velocity and ultimately translates into improved bioavailability of therapeutic candidates [43].

The comparison between *A. absinthium* CS- and NS-treatment groups indicated that NS at 100 mg/kg showed comparable hepatoprotective activity as 150 mg/kg of *A. absinthium* CS. The nanosuspension showed hepatoprotective potential at a smaller dose, which ultimately reduces side effects. The enhanced hepatoprotective activity of *A. absinthium* NS was due to improved bioavailability. The results agree with the study of Mishra et al. (2015), which demonstrated that nanosuspension formulation of *Phyllanthus amarus* (PA) extract showed better hepatoprotective potential than coarse extract. Nanoparticles of PA at a dose of 50 mg/kg were more effective than 125 mg/kg of PA coarse extract in providing hepatoprotection against paracetamol-induced hepatic injury. Devara et al. (2015) also reported that curcumin in the form of intravenous nanosuspension showed better liver protection as compared to the coarse suspension of the drug. Many other reports are also available on the formulation of nanosuspensions from medicinal plants such as *Azadirachta indica*, *Chrysanthemum coronarium* [44], and *Rauvolfia serpentina* [45], and the results reveal that nanoformulation has enhanced the bioavailability and potential of medicinal plants.

## 4. Conclusions

A nanosuspension of *Artemisia absinthium* extract with suitable particle size was successfully prepared. *Artemisia absinthium* nanosuspension is an appropriate pharmaceutical formulation with faster dissolution rate, enhanced bioavailability, and hepatoprotective ability. The study suggested that the prepared *Artemisia absinthium* nanosuspension is a potent hepatoprotective formulation having potential for commercialization in the pharmaceutical industry.

## Figures and Tables

**Table 1:** The effect of formulation factors on nanosuspension particle size, PDI, and zeta potential.

Formulation Code	Stabilizer-to-Plant Extract Ratio (*w*/*w*)	AS/S Ratio (*v*/*v*)	Stirring Time (Hours)	Particle Size (nm)	PDI	Zeta Potential (−mV)
A1	0.40	15.00	2.00	481.60	0.453	14.60
A2	0.70	18.41	4.00	277.00	0.295	11.40
A3	0.40	5.00	6.00	540.00	0.671	11.80
A4	0.70	10.00	0.64	578.00	0.682	9.70
A5	0.70	10.00	7.36	337.80	0.451	10.10
A6	1.00	15.00	6.00	250.00	0.285	13.10
A7	0.70	10.00	4.00	348.70	0.493	9.60
A8	0.20	10.00	4.00	572.20	0.536	16.50
A9	1.00	5.00	2.00	374.30	0.526	10.60
A10	1.00	15.00	2.00	417.60	0.503	9.70
A11	0.40	15.00	6.00	328.20	0.513	13.90
A12	0.70	10.00	4.00	352.00	0.481	12.50
A13	0.70	1.59	4.00	452.00	0.483	10.40
A14	0.40	5.00	2.00	656.20	0.718	14.20
A15	0.70	10.00	4.00	332.50	0.424	9.80
A16	0.70	10.00	4.00	358.00	0.416	10.30
A17	1.20	10.00	4.00	280.20	0.352	11.70
A18	0.70	10.00	4.00	342.00	0.468	11.40
A19	0.70	10.00	4.00	360.30	0.523	10.90
A20	1.00	5.00	6.00	271.00	0.242	9.80

As = antisolvent, S = solvent.

**Table 2:** Stability of *A. absinthium* nanosuspension at two different temperatures.

Storage Temperatures	Particle Size (nm)	PDI	Zeta Potential (mV)
25 °C	271.0 ± 0.02	0.706 ± 0.001	−10.6 ± 0.03
4 °C	286.6 ± 0.01	0.434±0.003	−9.22 ± 0.04

Every value is shown as the Mean ± SD (n = 3).

**Table 3:** Antioxidant activity of *A. absinthium* nanosuspension.

Treatments	IC50 Value (µg/mL)
A. absinthium nanosuspension	196.93 ± 0.13
A. absinthium extract	248.32 ± 0.21
Ascorbic acid	125.94 ± 021

Every value is shown as the Mean ± SD (n = 3).

**Table 4:** Effects of *A. absinthium* nanosuspension on hepatic biomarkers.

Hepatic Biomarkers	Normal Control G-I	Positive Control (CCl4) G-II	CCl4 + AA NS (100 mg/Kg)	CCl4 + AA CE (150 mg/Kg)
AST (IU/L)	56.00 ± 0.27	108.17 ± 0.67 #	56.31 ± 0.46 *	56.45 ± 0.20 *
ALT (IU/L)	29.43 ± 0.18	98.23 ± 0.45 #	29.27 ± 0.21 *	29.18 ± 0.44 *
ALP (IU/L)	164.47 ± 8.98	387.23 ± 5.32 #	242.39 ± 8.25 *	240.00 ± 7.25 *
Bilirubin (mg/dL)	0.54 ± 0.16	0.83 ± 0.12 #	0.52 ± 0.25 *	0.52 ± 0.27 *
Albumin (g/dL)	3.90 ± 0.28	2.61 ± 0.43 #	3.85 ± 0.26 *	3.85 ± 0.47 *
Total protein (g/dL)	9.15 ± 0.18	5.52 ± 0.33 #	8.78 ± 0.38 *	8.76 ± 0.20 *

Every value is shown as the Mean SEM (n = 4). AANS: # Significant difference (*p* < 0.05) from normal control group, * significant difference (*p* < 0.05) from positive control group. AA: *Artemisia absinthium,* NS: nanosuspension, CE: coarse extract.

**Table 5:** Effect of *A. absinthium* nanosuspensions on antioxidant enzymes.

Groups	GPx (IU/mg Protein)	CAT (IU/mg Protein)	SOD (IU/mg Protein)
Normal control	4.70 ± 0.18	42.21 ± 0.34	22.23 ± 0.25
Positive control	2.92 ± 0.32 #	20.49 ± 0.17 #	15.61 ± 0.20 #
AA CE (150 mg/kg)	4.39 ± 0.20 *	41.68 ± 0.34 *	20.49 ± 0.46 *
AA NS (100 mg/Kg)	4.01 ± 0.25 *	40.86 ± 0.22 *	20.78 ± 0.25 *

Results are shown as Mean SEM (n = 4) with significant differences (*p* < 0.05) between the normal control group and the positive control group. AA: *Artemisia absinthium,* NS: nanosuspension, CE: coarse extract, GPx: glutathione peroxidase, CAT: catalase, SOD: superoxide dismutase. # Significant difference (*p* < 0.05) from normal control group, * significant difference (*p* < 0.05) from positive control group. AA: *Artemisia absinthium,* NS: nanosuspension, CE: coarse extract.

## Data Availability

Not applicable.
